# Genome Sequence of the Heteropolysaccharide-Producing Strain Lactobacillus mucosae DPC 6426

**DOI:** 10.1128/genomeA.01350-14

**Published:** 2015-01-15

**Authors:** Paul M. Ryan, Caitriona M. Guinane, Lis E. E. London, Philip R. Kelleher, Gerald F. Fitzgerald, Noel M. Caplice, R. Paul Ross, Catherine Stanton

**Affiliations:** a Teagasc Food Research Centre, Moorepark, Fermoy, Co. Cork, Ireland; b School of Microbiology, University College Cork, Cork, Ireland; c Centre for Research in Vascular Biology, University College Cork, Cork, Ireland; d Alimentary Pharmabiotic Centre, Biosciences Institute, University College Cork, Cork, Ireland

## Abstract

Exopolysaccharide-synthesizing Lactobacillus mucosae DPC 6426 is a heterofermentative strain, which has demonstrated cholesterol-lowering properties in an animal model of lipid-driven atherosclerosis. The genome revealed a plethora of homologues linked to carbohydrate metabolism and mucin binding.

## GENOME ANNOUNCEMENT

Lactobacillus mucosae DPC 6426 is an exopolysaccharide (EPS)-producing strain originally isolated from the bovine gastrointestinal tract (1). L. mucosae DPC 6426 exhibits technological and biological robustness compared to a non-EPS-producing L. mucosae strain, indicating its versatility as an adjunct culture for functional food applications (1). Additionally, two recent studies have demonstrated the cholesterol-lowering effects of L. mucosae DPC 6426 in the atherosclerosis-prone apolipoprotein-E-deficient mouse model (2), as well as its application as an adjunct culture in yogurt for improving technofunctional properties (3).

The draft genome of L. mucosae DPC 6426 was sequenced by a combined paired-end 454 pyrosequencing on the FLX sequencer and an Illumina MiSeq approach by Beckman Coulter Genomics, with a final coverage of ~24× being achieved. The reads were assembled using the MIRA software (4) into 72 contigs. The order and orientation of the contigs were determined using the Artemis Comparison Tool (ACT) (5, 6) and progressiveMauve software (7) using the reference genomes of Lactobacillus fermentum IFO3956 (GenBank accession no. NC_010610), Lactobacillus reuteri JCM1112 (GenBank accession no. NC_010609), and L. mucosae LM1 (8). A combination of Glimmer 3.02, Prokaryotic Dynamic Programming Genefinding Algorithm (Prodigal) version 1.20 (http://prodigal.ornl.gov) (9, 10), and Rapid Annotations using Subsystems Technology (RAST) (http://rast.nmpdr.org/) (11) was used to identify and annotate predicted coding sequences (CDSs). The draft genome includes 2,079,103 bp, with an average G+C content of 46.7%. The genome consists of a single circular chromosome and does not appear to harbor plasmids. A total of 2,045 CDSs were predicted, including five rRNA operons, 47 tRNA genes, and a putative complete novel phage of 40.7 kb, Φ6426. A total GC-skew analysis and the open reading frame (ORF) orientation identified the *oriC* proximal to *dnaA* and the *terC* at ~1.06 Mb, and the coding density was predicted to be 85.4%.

The L. mucosae species was named as a result of an ability to adhere to intestinal mucosa, due to the apparent ubiquitous expression of mucus binding protein (*mub*) homologues within the species, as well as other adhesin-like proteins (12–14). The L. mucosae DPC 6426 genome was found to have predicted CDSs of mucin binding domains with similarities to the *mub* genes in L. mucosae and L. reuteri strains. Two putative collagen-binding proteins, a fibronectin binding protein (100% coverage and 70% identity to L. fermentum IFO3956 adherence protein), and five LPXTG motifs were also identified.

A 24-kbp putative EPS operon was identified, which codes for genes common to EPS-synthesizing lactobacilli, including those involved in regulation, chain length determination, repeating unit biosynthesis, and polymerization. Two additional regions (9.8 kbp and 6 kbp), flanked by putative insertion elements, were also identified as coding for putative glycosyltransferases, dextransucrases, and pectinsucrases.

The availability of the genome sequence of L. mucosae DPC 6426 will allow full analysis of the genetics behind its potential health-promoting attributes.

### Nucleotide sequence accession numbers.

This whole-genome shotgun project of L. mucosae DPC 6426 has been deposited at DDBJ/EMBL/GenBank under the accession number JSWI00000000. The version described here is JSWI01000000.
